# Poisson regression with adjustment for contamination and non-compliance in cohort studies conducted to estimate intervention effectiveness

**DOI:** 10.1177/09691413251388380

**Published:** 2025-11-10

**Authors:** Håkan Jonsson, Lennarth Nyström, Johannes Blom

**Affiliations:** 1 Department of Epidemiology and Global Health, 8075 Umeå University, Umeå, Sweden; 2 Department of Surgery, 27106 Södersjukhuset, Stockholm, Sweden; 3 Department of Clinical Science and Education, Karolinska Institutet, Stockholm, Sweden

**Keywords:** Cohort studies, contamination, non-compliance, rate ratio, Poisson regression

## Abstract

**Background:**

The effectiveness of an intervention such as cancer screening can be estimated by conducting a cohort study estimating the rate ratio between an exposed group invited to screening and a control group not invited to screening. A common issue is non-compliance, where not all individuals in the study group are exposed. Ignoring non-compliance can result in biased estimates of the exposure effect, but excluding non-exposed individuals may also be problematic as they may differ in risk profile from those who were exposed. A similar problem arises when members of the control group are inadvertently exposed (contamination). Observational studies face additional challenges due to confounding.

**Objective:**

To report the development of a method to adjust rate ratio estimates in cohort studies for contamination and non-compliance, that also adjusts for confounding.

**Method:**

Derivation of the new method is outlined.

**Results:**

The method is illustrated in two examples.

**Conclusion:**

The results are comparable with a stratified estimate, but through the use of a Poisson regression model the range of possible analyses is extended, for example to tests of confounding factors and interaction.

## Introduction

In cohort studies, individuals are followed over time and events such as diagnosis or death are recorded, and at follow-up outcome measures like rates are calculated. A common aim is to estimate the effect of an exposure by calculating a ratio of the outcomes, that is, rate ratio (RR), between an exposed study group offered a specific intervention, for example, cancer screening, and an unexposed control group. Exposure can be defined and measured in several ways but in the current study we only consider exposure as dichotomous, that is, exposed or non-exposed.

In experimental studies, randomization ensures that the study and control groups are similar except for the exposure, as random allocation tends to balance potential confounders, provided the sample size is sufficiently large. However, in observational studies confounding variables may be present and should ideally be adjusted for to avoid bias. For example, age is a confounder if it is a risk factor and is related to exposure, that is, it is differently distributed in the study group and the control group.

A common problem, in both randomized and observational studies, is when not all individuals in the study group are exposed, that is, *non-compliance.* An analysis that excludes non-exposed individuals is referred to as a *per-protocol analysis*, whereas an analysis that includes all participants regardless of exposure is called an *intention-to-treat analysis*. If there is an effect of the exposure, the intention-to-treat analysis is likely to underestimate the effect because non-exposed individuals are included in the study group. On the other hand, a per-protocol analysis may not necessarily provide an accurate estimate either as the non-exposed individuals in the study group might represent a selected group with a risk different from that of the average control group, leading to *selection bias*. Similar problems arise if there is an effect of the exposure and individuals in the control group are actually exposed, that is, *contamination*. Exposed individuals in the control group will underestimate the effect but selection bias in this group may also exist; therefore, excluding these individuals is not a solution. These problems have been described by Cuzick et al.^1^

It is common that non-compliance is the result of an individual choice not to participate in an offered intervention but there may be situations where non-exposure in the study group is not due to individual choice yet still reflects a non-random selection. In the current study, non-compliance refers to non-exposure in the study group, and contamination refers to exposure in the control group—regardless of whether it is due to individual choice or not.

Several methods have been used to adjust relative risks for non-compliance and contamination in randomized trials.^1,2^ To the best of our knowledge, comparable methods have not been developed for observational cohort studies with Poisson-distributed events that also account for confounding.

The aim of this study was to develop a method for cohort studies with Poisson-distributed events that adjust rate ratio estimates for contamination and/or non-compliance, while simultaneously accounting for confounding, using Poisson regression.

## Material and methods

### Adjustment for non-compliance

Below we derive how to estimate the rate ratio between an exposed study group and a non-exposed control group adjusted for non-compliance with events, for example, deaths, that are Poisson distributed, with expected values being a rate, for example, mortality, times the person-years. The estimate is derived in a similar way as for the outcome with binomial distribution by Cuzick et al.^1^

Let *c* be the control group, *s* the study group, and $s_{0},\;s_{1}$ the subgroups of non-compliers and compliers, respectively, in the study group, and with rates denoted as:

$\lambda_{c}$ is rate in control group (not exposed)
$\lambda_{s}$ is rate in study group
$\lambda_{s_{1}}$ is rate in compliers (exposed)
$\lambda_{s_{0}}$ is rate in non-compliers (not exposed).

The corresponding number of person-years and events are denoted $P_{i}$ and $O_{i}\;,\;i=c,s,s_{0},\;s_{1}$ , and the number of events follows a Poisson distribution with expected value $E(O_{i})=\lambda_{i}P_{i}$ .

Furthermore, let γ be the proportion of those who comply and $\lambda_{s_{1}}*$ the rate in compliers in study group assuming the exposure has no effect. An estimate of the true effect of the exposure is $\theta =\lambda_{s_{1}}/\lambda_{s_{1}}*$ , that is the actual rate in compliers divided by the rate we would have if the exposure had no effect, which adjusts for possible selection of those who comply.

The key step is the relationship $\lambda_{c}=\gamma \lambda_{s_{1}}*+(1-\gamma )\lambda_{s_{0}}=$ $\gamma \lambda_{s_{1}}/\theta +(1-\gamma )\lambda_{s_{0}}$ , which is solved as $\theta =\gamma \lambda_{s_{1}}/$ $\left(\lambda_{c}-(1-\gamma )\lambda_{s_{0}}\right)$ . An assumption for this relationship is that the proportion of non-compliers is the same in the control group as in the study group had they been offered the exposure, which is fulfilled in randomized trials but not obvious in observational studies.

The likelihood for the three groups is $\underset{i\in (c,s_{0},s_{1})}{\prod }\;(\lambda_{i}P_{i}{)}^{O_{i}}\exp(-\lambda_{i}P_{i})/O_{i}!$ . Maximizing gives the maximum likelihood (ML) estimates ${\hat{\lambda }}_{i}=O_{i}/P_{i},\;i=c,s_{0},\;s_{1}$ and since $P_{s_{1}}=\gamma P_{s}$ and $P_{s_{0}}=(1-\gamma )P_{s}$ we have $\hat{\theta }=(O_{s_{1}}/P_{s})/((O_{0}/P_{0})-(O_{s_{0}}/P_{s}))$ which is the ML estimate of the RR adjusted for non-compliance. We will however in the following express $\hat{\theta }$ as ${\mathrm{RR}}_{\mathrm{adj}}=((O_{s}-\alpha )/P_{s})/(O_{c}/P_{c}-\alpha /P_{s})$ where α is the number of events among non-compliers.

A more intuitive interpretation appears if we start from the intention-to-treat estimate ( α=0 ). Thus, $\mathrm{RR}=(O_{s}/P_{s})/(O_{c}/P_{c})$ which can alternatively be written $\mathrm{RR}=O_{s}/(O_{c}P_{s}/P_{c})$ where $O_{c}P_{s}/P_{c}$ can be interpreted as the number of events expected in the study group $\left(E_{s}\right)$ had they not been exposed. Thus, the estimate adjusted for non-compliance above can be written ${\mathrm{RR}}_{\mathrm{adj}}=(O_{s}-\alpha )/(O_{c}P_{s}/P_{c}-\alpha )=(O_{s}-\alpha )/$ $\left(E_{s}-\alpha \right)$ . Thus, if the number of events among non-compliers is subtracted from the total observed number of events in the study group, the same number has to be subtracted from the expected number of events.

### Adding adjustment for contamination

Expanding the estimate to include adjustment also for contamination is described below. We have for the control group $\lambda_{c}=\tau \lambda_{c_{1}}*+(1-\tau )\lambda_{c_{0}}$ where $\lambda_{c_{0}}$ is the rate in unexposed, $\lambda_{c_{1}}$ is the rate in contaminated (exposed), $\lambda_{c_{1}}*$ is the rate in the contaminated assuming the exposure has no effect and τ is the proportion contaminated. Thus, combining with the relationship above we have $\tau \lambda_{c_{1}}*+(1-\tau )\lambda_{c_{0}}=\gamma \lambda_{s_{1}}*+(1-\gamma )\lambda_{s_{0}}$ . We have $\theta =\lambda_{s_{1}}/\lambda_{s_{1}}*$ but also $\theta =\lambda_{c_{1}}/\lambda_{c_{1}}*$ , that is, the exposure effect is assumed to be the same in those who receive it. This means that $\tau (\lambda_{c_{1}}/\theta )+(1-\tau )\lambda_{c_{0}}=\gamma (\lambda_{s_{1}}/\theta )+(1-\gamma )\lambda_{s_{0}}$ . Solving this gives $\theta =(\gamma \lambda_{s_{1}}-\tau \lambda_{c_{1}})/((1-\tau )\lambda_{c_{0}}-(1-\gamma )\lambda_{s_{0}})$ and the ML estimate is $\hat{\theta }=(O_{s_{1}}/P_{s}-$ $O_{c_{1}}/P_{c})/(O_{c_{0}}/P_{c}-O_{s_{0}}/P_{s})$ , where $O_{c_{0}}$ and $O_{c_{1}}$ are the number of events in unexposed and exposed, respectively, in the control group. This can be written ${\mathrm{RR}}_{\mathrm{adj}}=$ $\left((O_{s}-\alpha )/P_{s}-\;\beta /P_{c})/((O_{c}-\beta )/P_{c}-\alpha /P_{s}\right)$ , where β is the number of events due to contamination, that is, events among the exposed in the control group. If there is no contamination ( β=0 ), we have the same ${\mathrm{RR}}_{\mathrm{adj}}$ adjusted only for non-compliance as above.

The rate ratio can be given a more intuitive interpretation: ${\mathrm{RR}}_{\mathrm{adj}}=(O_{s}-\alpha -\beta P_{s}/P_{c})/((O_{c}-\beta )P_{s}/P_{c}-\alpha )$ where $(O_{c}-\beta )P_{s}/P_{c}$ can be interpreted as the expected number of events among non-contaminated in the study group and $\beta P_{s}/P_{c}$ the expected number of contaminated events in the study group had it not been exposed. Thus, we have to subtract the expected number of events from the respective group to compensate for the removal of the actual events in non-compliers and contaminated.

### Poisson regression

To facilitate the use of a Poisson regression model for estimation, the ${\mathrm{RR}}_{\mathrm{adj}}$ above can be expressed as a ratio between two rates, ${\mathrm{RR}}_{\mathrm{adj}}=((O_{s}-\alpha -\beta P_{s}/P_{c})/P_{s})/((O_{c}-\beta -\alpha P_{c}/$ $P_{s})/P_{c})$ . We can then use the adjusted number of observations: $O_{s}-\alpha -\beta P_{s}/P_{c}$ as observation for the study group and $O_{c}-\beta -\alpha P_{c}/P_{s}$ as observation for the control group with offset based on the person-years $P_{s}$ and $P_{c}$ , respectively (offset in a log-linear Poisson model is the natural logarithm of the person-years). However, the numbers $\alpha P_{c}/P_{s}$ and $\beta P_{s}/P_{c}$ are most often not integers, but that is usually not a problem for maximization of the likelihood in standard software routines.

### Adjusting for covariates

In observational studies confounding may be present, so it is desirable to include covariates in the Poisson regression model. If there is a categorical covariate (i.e. a factor) that acts as a confounder, the method described can be used to adjust for non-compliance and contamination at each level of the factor separately. Thus, we have the observations $O_{si}-\alpha_{i}-\beta_{i}P_{si}/P_{ci}$ in the study group and $O_{ci}-\beta_{i}-\alpha_{i}P_{ci}/P_{si}$ in the control group with $P_{si}$ and $P_{ci}$ as basis for offsets, respectively, where i=1,2,…,k are the levels of the factor, see Example 1. By this, also different proportions of non-compliance and contamination between strata are adjusted for. With two or more factors, the same principle is used with the factor levels combined into strata; see Example 2 below.

### Variance adjustment

When we input the adjusted observations in the standard Poisson regression model the variance will be incorrect, so an adjustment of the variance is desirable. In a Poisson distribution the variance and the expected value are equal, but for the adjusted number of events in the study group $O_{s}-\alpha -\beta P_{s}/P_{c}$ this is not the case. Assuming that $O_{s}-\alpha$ and β are independent Poisson-distributed variables, the expected value is $E(O_{s}-\alpha )-E(\beta )P_{s}/P_{c}$ while the variance is $E(O_{s}-\alpha )+E(\beta )(P_{s}/P_{c}{)}^{2}$ , that is, larger than the expected value. The variance of the observation can however be adjusted by adding $\beta P_{s}/P_{c}(1+P_{s}/P_{c})$ to the Poisson variance. For the control group, the corresponding variance adjustment is $\alpha P_{c}/P_{s}(1+P_{c}/P_{s})$ . The statistical software R^3^ function “Poisson,” utilized for generating a Poisson family object, can be substituted with the slightly modified function “poisson_mod”, see Appendix (online), which incorporates the possibility to adjust the Poisson variance.

### Summary of the estimation steps

- The effect of exposure was estimated as the RR between the actual rate in exposed and the rate assuming the exposure had no effect. This exposure effect was assumed to be the same for all exposed, compliers as well as contaminated. Thereby the RR is adjusted for bias due to non-compliance and contamination.
- The adjusted RR was expressed as the ratio between two rates (adjusted number of events divided by person-years), where the events were assumed to follow a Poisson distribution with overdispersion, that is, a higher variance than in a standard Poisson distribution. The data can then be used in a Poisson regression model with an adjustment of the variance. A function for the variance adjustment for the software R is found in Appendix (online).
- By calculating the number of adjusted observed events and person-years in each stratum (combinations of covariate categories), Poisson regression with categorical covariates can be used to analyze the data and estimate the RR adjusted for confounding and possible differing proportions of non-compliance and contamination in strata.

### Prerequisites for the method

- Exposure in the study and control groups must be dichotomous, that is, exposed or not exposed. It is possible to categorize exposure as high or low, but this requires the additional assumption that all events within the same exposure category represent the same level of exposure.
- In each stratum (i.e. combination of factor levels) of the control group, the expected rate of potential non-exposed events is assumed to be the same as that for non-compliers (non-exposed) in the corresponding stratum of the study group, had they been offered the exposure. Similarly, if the study group had not been offered the exposure, the expected rate of potential contaminated events is assumed to match that of the contaminated (exposed) individuals in the control group.
- The numbers of correctly exposed events in the study group ( $O_{s}-\alpha$ ) and correctly non-exposed events in the control group ( $O_{c}-\beta$ ), as well as the number of events in non-compliers ( α ) and contaminated subjects ( β ), are assumed to follow a Poisson distribution.
- Covariates must be categorical factors.

### Stratified estimate

The strata-specific rate ratio estimates adjusted for non-compliance and/or contamination can be combined to a stratified estimate,^1^ ${\mathrm{RR}}_{\mathrm{strat}}=(1/\sum_{i}\;1/V_{i})\sum_{i}\;(1/V_{i}){\mathrm{RR}}_{\mathrm{adj},i}$ where ${\mathrm{RR}}_{\mathrm{adj},i}$ is the stratum-specific estimate and $V_{i}=V({\mathrm{RR}}_{\mathrm{adj},i})$ its variance. This estimate has been used for comparisons with the results from the Poisson model in the examples below since stratification will also adjust for confounding.

Calculations of confidence intervals for stratum-specific and stratified rate ratio estimates adjusted for non-compliance and contamination used in the examples below are shown in the Appendix (online). Wald's test was used for test of factors in the Poisson model.

## Results

To illustrate the method, we have created two examples estimating the effect of complying with invitation to an organized screening program for cancer. In the second example there is also contamination of the control group. Data have been manipulated so that adjustment for the factors age-group and sex is necessary to avoid bias due to confounding.

### Example 1: adjusting for non-compliance; one factor, age-group

The example has been developed to illustrate adjustment of non-compliance and one confounding factor. Assume that a study group (e.g. a geographical area) is invited to screening for a certain type of cancer, here called C, while the control group (another geographical area) is not invited. The aim of the screening is to prevent deaths caused by C, which is the outcome event in the study. There are two age-groups – young and old. The death rate due to C is higher in the old than in the young group (Table 1). In the study group there are twice as many person-years in the old than in the young, but in the control group the number of person-years in young and old are equal. Thus, there will be a bias due to confounding if the analysis is not adjusted for age-group. In the study group, there are non-exposed events (non-compliers) but there is no contamination in the control group. In the study group, there were 43 deaths from C among the younger and 128 among the older individuals. Of these, 17 and 50 deaths, respectively, occurred in individuals who were not exposed.

**Table 1.** Example 1: description of the study base.

Group	Outcome	Events	No. of events		
			Young	Old	Total
SG	PY × 1000		50	100	150
	Events	Total	43	128	171
		Non-exposed	17	50	67
		Adjusted	(43 − 17) = 26	(128 − 50) = 78	104
					
CG	PY × 1000		200	200	400
	Events	Total	206	309	515
		Exposed	0	0	0
		Adjusted	(206 − 17 × 200/50) = 138	(309 − 50 × 200/100) = 209	347

Number of person-years (PY) and events (total, non-exposed and adjusted) by age-group in the study group (SG) and the control group (CG).

**Figure 1.**
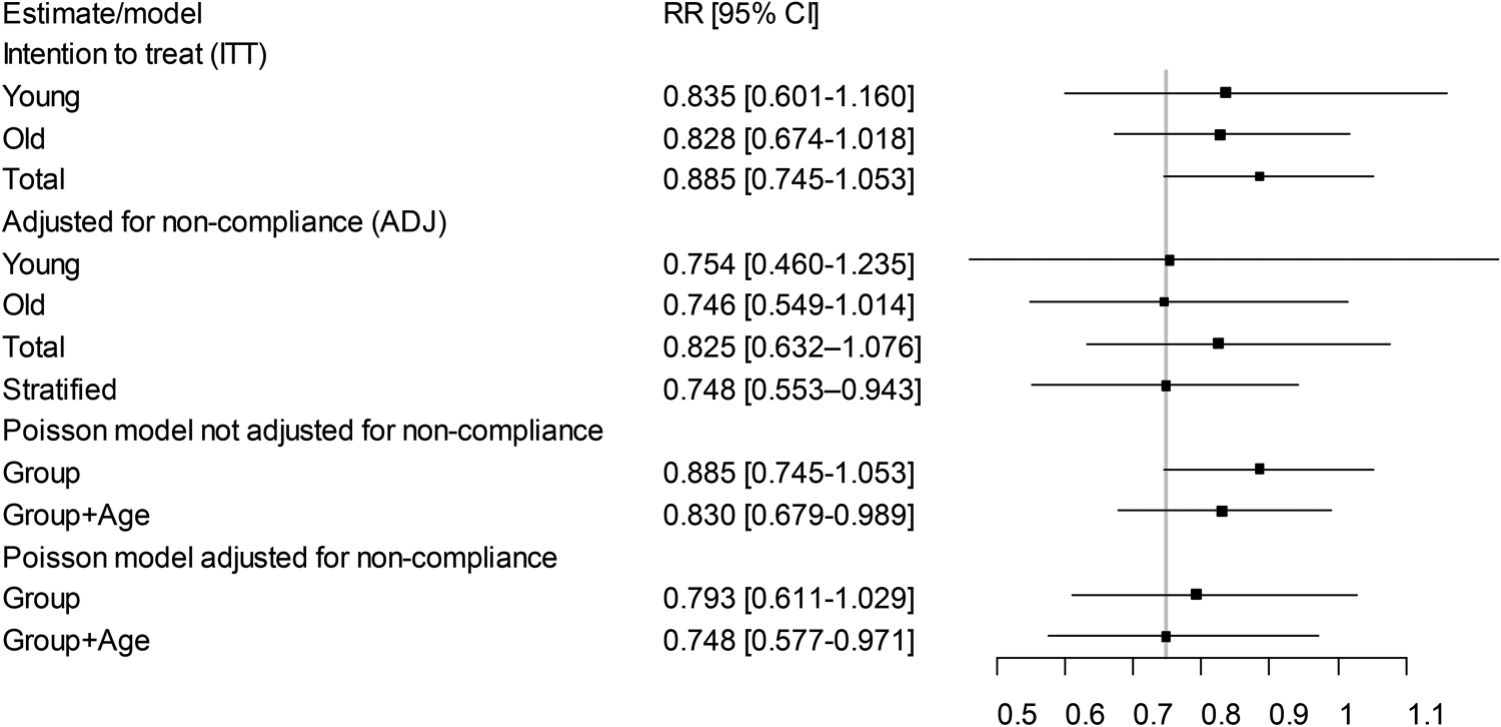
Estimates of rate ratio (RR) and 95% confidence intervals from Example 1. The Poisson models with group + age are adjusted for confounding together with the stratified estimate while the Poisson models with group only are not. Poisson model estimates adjusted for non-compliance are also variance adjusted. The vertical line illustrates the two RRs correctly adjusted for non-compliance and confounding (ADJ stratified and Poisson Group + Age).

Initially the stratum-specific and total (with observations summed across strata) RR estimates are presented for the intention-to-treat (ITT) analysis and adjusted for non-compliance (ADJ) analysis, followed by the stratified estimate (for ADJ only). Finally, estimates using Poisson models, with and without adjustment for non-compliance and variance, and including the covariates group, age-group, and their interaction, are presented. R code for the estimation is presented in Appendix (online). For the stratum-specific estimates the RR ITT demonstrates a lower effect than the RR ADJ (Table 2, Figure 1). Furthermore, the RR based on the total observations shows a lower effect than the stratum-specific estimates for ITT (0.885 [total] *vs*. 0.828 [old] and 0.835 [young]), as well as ADJ (0.825 [total] *vs*. 0.746 [old] and 0.754 [young]). The lower effect is due to confounding bias by age-group.

**Table 2.** Example 1: estimation of rate ratio (RR) and 95% confidence interval (CI) for comparison between the study and control group.

Model	Adjustments			Stratum	RR	95% CI
	Non-compliance	Confounding	Variance			
ITT						
Stratum-specific	No	–	–	Young	0.835	0.601–1.160
				Old	0.828	0.674–1.018
Total	No	No	–		0.885	0.745–1.053
ADJ						
Stratum-specific	Yes	–	–	Young	0.754	0.460–1.235
				Old	0.746	0.549–1.014
Total	Yes	No	–		0.825	0.632–1.076
Stratified	Yes	Yes	–		0.748	0.553–0.943
Poisson model						
Group	No	No	No		0.885	0.745–1.053
Group	Yes	No	No		0.799	0.642–0.995
Group	Yes	No	Yes		0.793	0.611–1.029
Group + Age	No	Yes	No		0.830	0.697–0.989
Group + Age	Yes	Yes	No		0.748	0.600–0.934
Group + Age	Yes	Yes	Yes		0.748	0.577–0.971
Group × Age-Group (interaction)	Yes	Yes	Yes	Young	0.754	0.460–1.235
				Old	0.746	0.549–1.014

Intention-to-treat (ITT), stratum-specific and total; adjusted for non-compliance (ADJ), stratum specific, total and stratified; and Poisson models with and without adjustment for non-compliance (Events) and for variance adjustment (Variance); models include the factors group and group + age and model with interaction terms.

The Poisson RR estimate not adjusted for non-compliance with group (study/control) only in the model is comparable to an ITT estimate and was in the example similar to the total ITT (RR 0.885). When adjusted for non-exposed events, RR decreased and further adjustment for variance widened the confidence interval (0.793; 95% CI 0.611–1.029). Adding the factor age-group, the unadjusted RR (ITT estimate but adjusted for age-group) was 0.830. Further, adjusting for non-compliance the RR became 0.748, which is the same as the stratified ADJ estimate. Also adjusting for variance, the 95% CI became, as expected, wider than with unadjusted variance (0.577–0.971) as compared to (0.600–0.934). The factor age-group was statistically significant (*p* = 0.005). The Poisson model with interaction between the two factors group and age-group resulted in similar estimates (0.746 and 0.754) and 95% confidence intervals for old and young as the stratum-specific ADJ RRs. Test of interaction between group and age-group was non-significant, *p* = 0.974 demonstrating that there is no evidence for a different effect of screening between old and young. This was expected since there was no interaction embedded in the data.

### Example 2: adjusting for both non-compliance and contamination; two factors, age-group and sex

Example 2 has the same setting as Example 1, but data are also split by sex (both age-group and sex are confounding factors) and there are events both due to non-compliance in the study group (not exposed) and contamination in the control group (exposed). The death rate due to C is higher in older than in younger and higher for men than for women (Table 3). In the control group the number of person-years in the four strata are similar, but in the study group the number of person-years is higher in older than in younger and higher in men than in women. Thus, there will be confounding due to age-group and sex. In this example all Poisson models were adjusted for non-compliance, contamination and variance.

**Table 3.** Example 2: description of the study base.

Group	Outcome	Events	No. of events				
			Men	Men	Women	Women	Total
			Young	Old	Young	Old	
SG	PY × 1000		120	230	100	150	600
	Events	Total	109	313	73	163	658
		Non-exposed	40	114	27	59	240
		Adjusted	109 − 40 − 35 × 120/200 = 48	313 − 114 − 53 × 230/200 = 138.1	73 − 27 − 28 × 100/200 = 32	163 − 59 − 42 × 150/200 = 72.5	658 − 240 − 158 × 600/800 = 299.5
CG	PY × 1000		200	200	200	200	800
	Events	Total	201	302	162	241	906
		Exposed	35	53	28	42	158
		Adjusted	201 − 35 − 40 × 200/120 = 99.3	302 − 53 − 114 × 200/230 = 149.9	162 − 28 − 27 × 200/100 = 80	241 − 42 − 59 × 200/150 = 120.3	906 − 158 − 240 × 800/600 = 428

Number of person-years (PY) and events (total, non-exposed, exposed, and adjusted) by age-group and sex in the study group (SG) and the control group (CG).

Initially the stratum-specific and total RR estimates are presented for both the ITT and ADJ analyses, followed by the stratified estimate (for ADJ only). Finally, estimates from Poisson models with covariates group, age-group, sex and interactions in the model are shown. The overall RR patterns are similar to those in Example 1 with larger effect when non-compliance and contamination were adjusted for (Table 4). For the totals, the RR estimates show a lower effect compared to the stratum-specific estimates due to confounding bias.

**Table 4.** Example 2: estimation of rate ratio (RR) and 95% confidence interval (CI) for comparison between the study and control group.

		Adjustments				Test	
Model	Stratum	Non-compliance and contamination	Confounding	RR	95% CI	Factor	*p*-Value
ITT							
Stratum-specific	Men, young	No	–	0.904	0.716–1.141		
	Men, old	No	–	0.901	0.769–1.056		
	Women, young	No	–	0.901	0.684–1.188		
	Women, old	No	–	0.902	0.739–1.100		
Total		No	No	0.968	0.876–1.071		
ADJ							
Stratum-specific	Men, young	Yes	–	0.805	0.491–1.320		
	Men, old	Yes	–	0.801	0.574–1.119		
	Women, young	Yes	–	0.800	0.445–1.438		
	Women, old	Yes	–	0.803	0.529–1.219		
Total		Yes	No	0.933	0.752–1.157		
Stratified		Yes	Yes	0.802	0.630–0.974		
Poisson model							
Group		Yes	No	0.837	0.674–1.039		
Group + Age		Yes	Partial	0.811	0.654–1.006	Age	0.0004
Group + Sex		Yes	Partial	0.828	0.667–1.028	Sex	0.042
Group + Age + Sex		Yes	Yes	0.802	0.647–0.994	Sex	0.050
						Age	0.0004
Adding two-way interactions to Group + Age + Sex		Yes	Yes			Group × Age	0.995
						Group × Sex	1.000
						Age × Sex	0.996

Intention-to-treat (ITT), stratum-specific and total estimates; adjusted for non-compliance and contamination (ADJ), stratum-specific, total and stratified; and Poisson models with adjustment for non-compliance and contamination and adjusted variance; models include the factors group, group + age, group + sex and group + age + sex, and models with interaction terms.

The Poisson model adjusted for both age-group and sex yielded a RR of 0.802 (95% CI, 0.647–0.994), which is similar to the stratified RR estimate adjusted for non-compliance and contamination (ADJ). Age-group was statistically significant (*p* = 0.0004) in the model while sex was borderline significant (*p* = 0.050). The two-way interactions were also tested as added to the former model but were not significant, that is, no evidence for different effect of screening between young and old or between men and women. This is not surprising since there was no interaction embedded in the data.

## Discussion

We have developed a new method to adjust RRs for non-compliance and contamination for Poisson-distributed events in cohort studies with adjustment for confounding using Poisson regression analysis. The method facilitates an extended number of statistical analyses compared to a stratified analysis.

In observational studies on the effectiveness of mammography screening, methods to adjust for non-compliance have been applied by estimating RR for non-attenders.^4,5^ Moreover, the crude ML estimate in the present paper has previously been applied to adjust for non-exposed cases (not invited),^6,7^ as well as for both non-invited individuals and non-participants.^8,9^

### Strengths

Our method can be applied to adjust for non-compliance, contamination and confounding in cohort studies. The use of a Poisson regression model facilitates analysis and testing of confounding factors and interactions, for example, whether the effect differs between age-groups, as well as estimation and testing of trends by using the covariate factor as a continuous variable. Hierarchical models, that is, models with different number of parameters, can also be compared using a likelihood ratio test. Poisson regression is an established and accessible statistical method and the software used for the estimation, R,^3^ is a freeware.

One advantage with this method is that it does not require the calculation of RRs for subgroups (e.g. non-participants), or the probabilities of being exposed given the allocated group. Exposure information is only required for the events, not on all individuals. For instance, in Example 1 within the study group there is an unknown number of non-exposed individuals among the young, but the calculation requires only the number of events among non-exposed (17) and the total number of events (43) along with the overall number of person-years (50,000).

The method is also applicable to situations where the proportions of non-compliance and/or contamination differ across strata, for example, a lower proportion of non-compliance in young than in old.

### Limitations

One limitation of the method is that if there are several factors, each with many categories, the total number of strata (i.e. combinations of factor levels) can become large. For example, four factors with five categories each result in 625 possible strata. If the number of strata is large relative to the total number of events, each stratum will, on average, contain few observations. By chance, the adjusted number of events in some cells may become negative. Since negative event counts cannot be handled, they must be set to zero, introducing a residual adjustment. We therefore recommend avoiding unnecessary factors and reducing the number of levels wherever possible. However, if the number of residual adjustment events is small relative to the total number of adjusted events required, the resulting bias will be minimal.

A similar problem can arise when there is an imbalance between the study group and the control group. For example, if non-exposed events in a stratum of the study group cannot be adjusted for, due to the absence of a matching stratum in the control group, the adjustment cannot be performed. A pragmatic solution is to redistribute the residual adjustments to neighboring strata. The effectiveness of this approach may depend on how the factors relate to non-compliance and/or contamination. For instance, if non-compliance varies by age-group but not by sex, it may be acceptable to reassign the stratum for adjustment from men to women within the same age-group without introducing bias—but not the other way around.

### Overdispersion

The adjusted observations have a higher variance then expected value, that is, overdispersion.^10^ Unlike quasi-likelihood models, where overdispersion is estimated, the variance in the current model is adjusted. While quasi-likelihood models are robust, the proposed method is also expected to perform well.

### Exposure in cancer screening

In organized cancer screening for early detection, that is, secondary prevention, the exposure ‘participation in the screening program’ can be defined with respect to time of the cancer diagnosis. This is because the exposure is relevant only during the period in which early-stage cancer can be detected. A common definition of being exposed is that the individual must have participated in the most recent screening round preceding the cancer diagnosis. For example, consider an individual who participated in a cancer screening program that invited individuals aged 50–69 to biennial screenings, and who died from the disease at age 76, having been diagnosed with cancer at age 73 that is, after the screening program had ended. Thus, even if the individual participated in all rounds up to age 69, at the time of diagnosis at least four years had passed since the last screening participation, so this person is not considered exposed.

### Individual choice

Non-compliance is usually regarded as an individual choice not to participate in an offered intervention and may therefore represent a selected group. However, there may be situations where non-exposure in the study group is not due to individual choice yet still reflects a non-random selection. In the example above, the individual was diagnosed at age 73, that is, 4 years after the screening invitations had ended, and was classified as non-exposed. This classification was not a matter of individual choice; nevertheless, a systematic bias toward non-exposure at older ages persists in such cases.

## Conclusions

When using cohort studies to evaluate the effectiveness of an intervention it is important to adjust for non-compliance and contamination while also accounting for confounding factors. The proposed method, using Poisson regression, provides unbiased estimates in settings involving non-compliance, contamination and confounding, as demonstrated in the two examples. The resulting estimates in the examples were similar to the stratified method. Further, the use of the Poisson regression model extends the range of possible statistical analyses.

## Supplemental Material

sj-pdf-1-msc-10.1177_09691413251388380 - Supplemental material for Poisson regression with adjustment for contamination and non-compliance in cohort studies conducted to estimate intervention effectiveness Supplemental material, sj-pdf-1-msc-10.1177_09691413251388380 for Poisson regression with adjustment for contamination and non-compliance in cohort studies conducted to estimate intervention effectiveness by Håkan Jonsson, Lennarth Nyström and Johannes Blom in Journal of Medical Screening
