# Supplementary material for: Poisson regression with adjustment for contamination and non-compliance in cohort studies conducted to estimate intervention effectiveness
Source: J Med Screen. 2025 Nov 10;33(2):71–8. doi: 10.1177/09691413251388380 (PMC13201887; doi:10.1177/09691413251388380)
Supplement: sj-pdf-1-msc-10.1177_09691413251388380 - Supplemental material for Poisson regression with adjustment for contamination and non-compliance in cohort studies conducted to estimate intervention effectiveness [file sj-pdf-1-msc-10.1177_09691413251388380.pdf]

Appendix

Example using poisson_mod

A general example of an R command for analysis using "poisson_mod" is

glm(obs_adj~group+age,offset=log(P),family=poisson_mod(o=df$vadd),data=df),

where df is a data frame with the variables; obs_adj (the adjusted number of events), group (study or control), age (a factor e.g. young or old), P (person-years) and vadd (added variance adjustment) which is $\beta\, P_s/P_c\,(1+P_s/P_c)$ for the study group and $\alpha\, P_c/P_s\,(1+P_c/P_s)$ for the control group.

Code used for estimation in Example 1

Below find R code for estimation of the Poisson model in Example 1 adjusted for non-compliance and confounding (covariate factor age) and with adjusted variance. The variance adjustments were given in the input data to simplify the code.

```
#  Package used for "ci.exp" below
library(Epi)

#  Create a data frame
df_ex1<-data.frame(group=factor(c(1,1,0,0)),age=factor(c("young","old","young","old")),P=c(50,100,200,200),
              O=c(43,128,206,309),obs_adj=c(26,78,138,209),vadd=c(0,0,340,300))

# The variance adjustments are
#
# Study group
# vadd = 0 since β = 0 (no contamination)
#
# Control group
#  vadd (young) = 17*200/50*(1+200/50) = 340
#  vadd (old) = 50*200/100*(1+200/100) = 300

# Poisson model estimation using poisson_mod
res<-glm(obs_adj~group+age,offset=log(P),family= poisson_mod (o=df_ex1$vadd),data=df_ex1)

# Show results
summary(res)
# Adjusted rate ratio and 95% confidence intervals
round(ci.exp(res),3)[2,]
```

Modified Poisson family object "poisson_mod"

The suggested family object (poisson_mod), where the variance function in the Poisson family object is modified by o=var.adj can be created in R using the code below

```
poisson_mod<-function (o=var.adj,link = "log")
{
  linktemp <- substitute(link)
```

```
  if (!is.character(linktemp))
    linktemp <- deparse(linktemp)
  okLinks <- c("log", "identity", "sqrt")
  family <- "poisson"
  if (linktemp %in% okLinks)
    stats <- make.link(linktemp)
  else if (is.character(link)) {
    stats <- make.link(link)
    linktemp <- link
  }
  else {
    if (inherits(link, "link-glm")) {
      stats <- link
      if (!is.null(stats$name))
        linktemp <- stats$name
    }
    else {
      stop(gettextf("link \"%s\" not available for %s family; available links are %s",
                    linktemp, family, paste(sQuote(okLinks), collapse = ", ")),
           domain = NA)
    }
  }
  # Modified variance function
  variance <- function(mu) mu+o
  validmu <- function(mu) all(is.finite(mu)) && all(mu > 0)
  dev.resids <- function(y, mu, wt) {
    r <- mu * wt
    p <- which(y > 0)
    r[p] <- (wt * (y * log(y/mu) - (y - mu)))[p]
    2 * r
  }
  aic <- function(y, n, mu, wt, dev) -2 * sum(dpois(y, mu,
                                                   log = TRUE) * wt)
  initialize <- expression({
    if (any(y < 0)) stop("negative values not allowed for the 'Poisson' family")
    n <- rep.int(1, nobs)
    mustart <- y + 0.1
  })
  simfun <- function(object, nsim) {
    wts <- object$prior.weights
    if (any(wts != 1))
      warning("ignoring prior weights")
    ftd <- fitted(object)
    rpois(nsim * length(ftd), ftd)
  }
  structure(list(family = family, link = linktemp, linkfun = stats$linkfun,
                 linkinv = stats$linkinv, variance = variance, dev.resids = dev.resids,
                 aic = aic, mu.eta = stats$mu.eta, initialize = initialize,
                 validmu = validmu, valideta = stats$valideta, simulate = simfun),
            class = "family")
}
```

Confidence interval for $log(RR_{adj})$

Approximation of the variance for $log(RR_{adj})$ can be calculated using the mean value theorem (i.e., first order approximation of a Taylor series) where $V(log(x)) \approx \frac{V(x)}{E(x)^2}$.

$$V\left(log(RR_{adj})\right) = V\left(log\left(\frac{(O_s - \alpha - \beta\, P_s/P_c)/P_s}{(O_c - \beta - \alpha\, P_c/P_s)/P_c}\right)\right)$$

$$= V(\log((O_s - \alpha - \beta\, P_s/P_c)/P_s)) + V(\log((O_c - \beta - \alpha\, P_c/P_s)/P_c))$$
$$+ V(-log(P_s)) + V(-log(P_c))$$
$$= V(\log(O_s - \alpha - \beta\, P_s/P_c)) + V(\log(O_c - \beta - \alpha\, P_c/P_s))$$
$$\approx \frac{V(O_s - \alpha - \beta\, P_s/P_c)}{E(O_s - \alpha - \beta\, P_s/P_c)^2} + \frac{V(O_c - \beta - \alpha\, P_c/P_s)}{E(O_c - \beta - \alpha\, P_c/P_s)^2} \,\widehat{=}\, \frac{O_s - \alpha + \beta(P_s/P_c)^2}{(O_s - \alpha - \beta\, P_s/P_c)^2}$$
$$+ \frac{O_c - \beta + \alpha(P_c/P_s)^2}{(O_c - \beta - \alpha P_c/P_s)^2}$$

To calculate a 95% confidence interval $exp\left(log(RR_{adj}) \pm 1.96\sqrt{V\left(log(RR_{adj})\right)}\right)$ was used assuming the estimate follows a standard normal distribution.

Confidence interval for the stratified estimate

The stratified estimate according to Cuzick et al. is $RR_{strat} = \frac{1}{\sum_i \frac{1}{V_i}} \sum_i \frac{1}{V_i} RR_{adj,i}$ where $V_i = V(RR_{adj,i})$.

V_i can be calculated by using the approximation $V(RR_{adj}) \approx V\left(log(RR_{adj})\right) E(RR_{adj})^2$. Thus

$$V(RR_{strat}) = \left(\frac{1}{\sum_i \frac{1}{V_i}}\right)^2 \sum_i \frac{1}{V_i^2} V(RR_{adj,i}) = \left(\frac{1}{\sum_i \frac{1}{V_i}}\right)^2 \sum_i \frac{1}{V_i} = \frac{1}{\sum_i \frac{1}{V_i}}$$

To calculate a 95% confidence interval we have used $RR_{strat} \pm 1.96\sqrt{V(RR_{strat})}$ assuming the estimate follows a standard normal distribution.
